# Characterization of epidemiological distribution and outcome of COVID-19 in patients with hereditary hemorrhagic telangiectasia: a nationwide retrospective multi-centre study during first wave in Italy

**DOI:** 10.1186/s13023-021-02000-2

**Published:** 2021-09-08

**Authors:** Patrizia Suppressa, Fabio Pagella, Gennaro Mariano Lenato, Eleonora Gaetani, Ilaria Serio, Maristella Salvatora Masala, Giuseppe Spinozzi, Roberta Lizzio, Elina Matti, Annalisa De Silvestri, Giulio Cesare Passali, Maria Aguglia, Claudia Crocione, Carlo Sabbà

**Affiliations:** 1 DIM-Interdisciplinary Department of Medicine, “Frugoni” Internal Medicine and Geriatrics Unit, HHT Interdepartmental Center, VascERN HHT Reference Center, Policlinico Hospital, University of Bari, P.zza Giulio Cesare, 70124 Bari, Italy; 2 Department of Otorhinolaryngology, Fondazione IRCCS Policlinico San Matteo, Pavia, Italy; 3 University of Pavia, Pavia, Italy; 4 Department of Medical and Surgical Sciences, Multidisciplinary Gemelli Group for HHT, Fondazione Policlinico Universitario Agostino Gemelli IRCCS, Università Cattolica del Sacro Cuore School of Medicine, Rome, Italy; 5 Division of Internal Medicine, Department of Medical and Surgical Sciences, IRCCS Azienda Ospedaliero-Universitaria di Bologna, University of Bologna, Bologna, Italy; 6 Clinica Medica Unit, Sassari University Hospital, Sassari, Italy; 7 Clinical Epidemiology and Biometry Unit, Fondazione IRCCS Policlinico San Matteo, Pavia, Italy; 8 Division of Otorhinolaryngology, Multidisciplinary Gemelli Group for HHT, Fondazione Policlinico Universitario Agostino Gemelli IRCCS, Università Cattolica del Sacro Cuore School of Medicine, Rome, Italy; 9 Clinical Pathology Unit, Vito Fazzi Hospital, Lecce, Italy; 10 HHT Onlus Patient Association, Rome, Italy

**Keywords:** Hereditary hemorrhagic telangiectasia, Rare diseases, COVID-19, PANDEMICS 2020, SARS-CoV-2 infection

## Abstract

**Background:**

Coronavirus Disease 2019 (COVID-19) continues to have a devastating impact across the world. A number of pre-existing common clinical conditions were reported to represent risk factors for more severe COVID-19 outcomes. Hereditary Hemorrhagic Telangiectasia (HHT) is a rare vascular heritable disorders, characterized by complications secondary to visceral Arterio-Venous Malformations. The impact of HHT, as well as for many Rare Diseases (RDs) on infection susceptibility profile and clinical adverse outcome risk is an unresolved issue.

**Objectives:**

The main objectives were: to assess the clinical features and outcomes of HHT patients infected with COVID-19; to compare the relative infection risk in these patients with the Italian general population throughout the first pandemic wave; to investigate the factors potentially associated with severe COVID-19 outcome in HHT patients, and the possible impact of COVID-19 infection on HHT-related symptoms/complications. Finally, we aimed to estimate how the lockdown-associated wearing of personal protective equipment/individual protection devices could affect HHT-related telangiectasia bleeding frequency.

**Methods:**

The study is a nation-wide questionnaire-based survey, with a multi-Center retrospective cross-sectional design, addressed to the whole Italian HHT population. COVID-19 cases, occurring throughout the first pandemic wave, were collected by a questionnaire-based semi-structured interview. Only the cases ascertained by laboratory confirmation (molecular/serological) were included for epidemiological estimates. Information concerning eventual SarS-Cov-2 infection, as well as regarding HHT-related manifestations and HHT-unrelated co-morbidities were collected by the questionnaire. Prevalence data were compared to Italian general population in the same period.

**Results:**

The survey disclosed 9/296 (3.04%) COVID-19 cases, 8/9 of them being resident in Lombardy, the main epidemic epicenter. Pneumonia was reported by 4/9 patients, which prompted hospital admission and intensive care management in 2 cases. No fatal outcome was recorded. After careful refinement of epidemiological analysis, the survey evidenced overlapping infection risk in HHT compared to general population.

**Conclusions:**

COVID-19 infection profile parallels geographical distribution of epidemic foci. COVID-19 in HHT patients can lead to highly variable clinical profile, likely overlapping with that of general population. The HHT disease does not seem to involve a different approach in terms of hospital admission and access to intensive care with respect to general population.

## Introduction

Coronavirus Disease 2019 (COVID-19), caused by the Severe Acute Respiratory Syndrome coronavirus 2 (SarS-CoV-2), was first described in December 2019 in Wuhan, China, rapidly raising international concern due to its high burden of contagiousness, morbidity, and mortality [1]. COVID-19 shows a wide range of clinical manifestations [2]. Whilst showing in most cases completely absent or mild symptoms, COVID-19 disease can also entail hospitalization, progression into respiratory distress and death [3, 4]. Old age and several pre-existing common clinical conditions, such as cardiovascular diseases, chronic pulmonary diseases, obesity, and diabetes mellitus, were reported to be prominent risk factors for severe COVID-19 outcomes [5]. On the other hand, data regarding the impact of specific Rare Diseases (RDs) on infection susceptibility and clinical adverse outcome risk are still poorly elucidated, and often limited to small case series and expert opinions [6].

Despite patients with RDs typically represent vulnerable populations [7], data collection on these cohorts have thus-far been precluded by the inherent and well-known difficulties in RD-involving research studies [8], as well as by fast spread of SarS-Cov-2 infection [9]. As a consequence, insufficient evidence is currently available to guide clinicians in the management of these patients, as well as to implement healthcare policy-making towards optimization of patients’ pathways and infection prevention strategies [6, 10].

Hereditary Hemorrhagic Telangiectasia (HHT) is a rare disease inherited as an autosomal dominant trait and characterized by multiple localized angiodysplasia [11, 12]. The majority of HHT-causing variants are detected in endoglin (*ENG*) and activin A receptor type II-like kinase 1 (*ALK1/ACVRL1*) genes [13]. Loss-of-function variants result in excessive endothelial proliferation and impaired angiogenic remodeling, ultimately leading to abnormal arterio-venous connections. [14]. Hence, clinical hallmarks of HHT disease are multiple arterio-venous shunts, or arterio-venous malformations (AVMs), affecting several organs, such as nasal and oral mucosa, skin, lung, liver, brain, and GI tract [15, 16]. Although the commonest symptom is represented by spontaneous, repeated nosebleeds, affecting over 90% of patients [17], visceral involvement may entail sudden AVM-related complications [18, 19].

Due to its multi-systemic involvement, the presence of underlying HHT condition might modify the COVID-19 infection susceptibility profile, as well as the COVID-19 disease course in infected individuals, with many respects. Due to the very high prevalence of spontaneous and recalcitrant nosebleeds, HHT patients are subjected to frequent self-manipulation of nostrils, nasal cavity, or nasopharynx, with subsequent augmented risk to SarS-Cov-2 exposure [20]. Furthermore, HHT patients may experience episodes of emergency-care-requiring massive epistaxis, as well as other complications secondary to visceral AVMs, which require prompt access to hospital setting and frequent contact with medical personnel, thus resulting in consequent increased risk of contracting the infection [21–23].

Additionally, many of HHT-related manifestations are thought to be potentially associated with a worst clinical course, such as chronic anoemia, dyspnea, brain abscess/stroke, intracranial haemorrhage, portal hypertension, high-output heart failure, pulmonary hypertension, GI bleeding [20]. However, no specific data are available about the risk of a SarS-CoV-2 infection in patients previously diagnosed with HHT. In addition, in order to standardize tailored therapeutic protocols, data regarding the clinical/healthcare trajectory of COVID-19 subjects with underlying HHT condition are urgently needed.

Finally, several studies reported that HHT is associated to a considerably impaired quality of life [24, 25]. This scenario may even more be exacerbated in Italy during the first wave, in light of the restrictive measures which were applied to avoid the collapse of the national health system in front of the devastating and unexpected spreading of COVID-19 [26]. Uncontrollable epidemic spreading induced Italian Government to declare a nationwide lockdown and to release indication for mandatory utilize of personal protective equipment/individual protection devices (PPE/DPI) [27]. On the other hand, prolonged wearing of face and hand covering tools might have affected bleeding frequency of nasal, oral, and skin telangiectases in HHT patients.

To better define the factors underlying the characteristics of COVID-19 infection among HHT patients, we carried out a nationwide epidemiological multi-center study. Our objectives were to assess the clinical features and outcomes of HHT patients infected with COVID-19 and to compare the relative infection risk in these patients with the Italian general population throughout the first wave. We also tried to investigate the factors potentially associated with severe COVID-19 outcome in HHT patients, and the possible impact of COVID-19 infection on HHT-related symptoms/complications. Finally, we aimed to estimate how the lockdown-associated PPE/DPI wearing could affect HHT-related telangiectasia bleeding frequency.

## Methods

### Design

The study is a nation-wide questionnaire-based survey, with a multi-Center retrospective cross-sectional design, addressed to the whole Italian HHT population. Patients were recruited among Italian subjects with HHT through the existing network of 5 Italian HHT Centers of expertise (Bari University “Policlinico” Hospital VascERN Center, Pavia Fondazione IRCCS “Policlinico San Matteo” University Hospital, Rome Fondazione IRCCS “Policlinico Gemelli” Catholic University Hospital, Bologna IRCCS University Hospital, Sassari University Hospital). Patients with a genetically and/or clinically confirmed diagnosis of HHT [18, 28–30] who were currently in follow-up at one of the 5 participating HHT Centers were considered to be eligible for the present study, and were contacted by telephone/email. To minimize any possible bias, patients (or parents in case of minors) were contacted with no selection criteria, such as age, gender, education and marital status, and independent of clinical/manifestations. To catch eventual information regarding individual with COVID-19-related fatal outcome, close-relationship familiars or caregivers of HHT patients were also contacted in some cases. Prior to questionnaire administration, explicit informed consent to participate to the study was released by the respondent. The study was approved by the Ethics Committee of all of the 5 participating Institutions.

### Questionnaire

The questionnaire was a semi-structured interview composed of two sections. The first section included information concerning eventual SarS-Cov-2 infection, such as symptom onset (Fever, Coughing, Headache, Dyspnea, Muscular Pain, Sense of Tiredness/Fatigue, Diarrhoea, Sore Throat, Rhinorrhea, Conjunctivitis, Gustative/olfactory sensory impairment, Asymptomatic), diagnostic procedures, clinical course, final outcomes (resolution, need for hospitalization, post-discharge sequelae, death). Summary measures regarding infection-related clinical manifestations included both general COVID-19-associated symptoms and more specific HHT-typical features, concerning bleeding profile change and sense of dryness/discomfort of the nasal and oral mucosa. Specific questions distinguished ascertained COVID-19 cases, based on laboratory test confirming molecular and/or serological diagnosis (namely, SarS-Cov-2-specific Polymerase Chain Reaction (PCR)-based molecular assay on Nasopharyngeal/Oropharyngeal Swab [31], and/or anti-SarS-Cov-2 Ab serological test [32], respectively), from suspected cases, characterized by the subjective reporting of COVID-19-suggestive symptoms lacking molecular/serological confirmation. Since the main objective of the study was focused on the first pandemic wave, only the cases occurring in this study period were included (2020, January 1st–June 30th).

The second section collected general data and baseline clinical information, regarding the province of residency throughout the lockdown period, site of HHT-related AVM involvement, presence of common HHT-unrelated comorbidities, details of possible telangiectasia bleeding associated to wearing of PPE/DPI.

### Epidemiology

COVID-19 prevalence rates in HHT were calculated as observed number of COVID-19 cases during the study period divided by the total number of respondents to the study. For sake of epidemiological estimates, only cases with ascertained SarS-Cov-2 infection diagnosis were considered. To account for the dramatic prevalence dishomogeneity that characterized the first pandemic wave in Italy [33], we obtained prevalence rates for single Regions, based on the patient’s actual residency throughout lockdown. Prevalence data in HHT were compared with Italian general population, by considering the number of COVID-19 cases reported in each Italian Region/Province on 2020 June 30th, by official Italian Ministry of Health reports [34].

A further refinement of epidemiological estimates was achieved by targeting possible bias in respondent’s population composition, as it is plausible that respondents can be enriched with individuals with ascertained and/or suspected COVID-19 infection. Hence, we decided to compute prevalence rates through expanding the denominator of susceptible individuals, by considering all the patients eligible to the present study who were contacted by one of the participating Center, and not only the respondents.

Thirdly, to optimize homogeneity between our population and Italian general population, we repeated the calculation, by restricting the analysis of single-region epidemiological comparison only to those patients whose diagnosis had been confirmed by PCR-based molecular assay on Nasopharyngeal/Oropharyngeal Swab, and excluding those patients with only serological positive test. Such restriction was needed to permit a proper comparison between the study population and the reference population, given that our inquiry was designed to catch all laboratory-test-confirmed COVID-19 patients (molecular and/or serological confirmation), whereas the general population’s point prevalence data in Italy did not include serological-test-positive individuals lacking molecular assay confirmation, in the first wave [32].

### Statistical analysis

Quantitative variables are described as means and standard deviation; categorical variables are reported as count and percentage. For each variable, missing data were excluded from analysis. Prevalence of SarS-Cov-2 infection is reported with 95% confidence interval (95% CI). Comparisons with general population are made with chi square test. Relative risk (RR) calculated as prevalence ratio with 95% CI is reported.

### Patient involvement

Patients advocates from HHT Onlus were involved in the design and conduct of this research project. Priorities of the research, choice of primary and secondary outcome measures, and methods of recruitment were informed by discussions with patients delegates of HHT Onlus that took part in all study development meetings. As results emerged they were reviewed with the patient advocates collecting their perspectives and feedback to ensure that we presented the findings in the most effective way to the general population. This method allowed us to collect valuable contributions from patient advocates during the entire study process while the investigators retained the rigors of scientific work. Guarantee of dissemination of findings to the general population will be ensured also through collaboration with HHT Onlus advocacy activities.

## Results

### Baseline clinical features

A total of 296 HHT patients responded to the questionnaire (mean age 52.94 ± 17.59 years, range 3–86 years, female gender ratio = 157/296, 53.0%). Baseline HHT-related and -unrelated clinical features of respondent patients are shown in Table 1. A mutation in a known disease-causing gene was identified in 236/296 (79.7%) patients, while the mutational analysis had been inconclusive or was still ongoing in the remaining 60/296 (20.3%) patients. Among patients with identified mutation, 86/236 (36.5%) patients were carriers of an *ENG* mutation (HHT type 1), 149/236 (63.1%) patients were carriers of an *ALK1/ACVRL1* mutation (HHT type 2), while 1/236 (0.42%) patient belonged to the SMAD4-HHT type. All of the 60 mutation-negative patients had a certain clinical diagnosis of HHT, according to Curaçao criteria. Furthermore, patients were also asked to report co-morbid conditions, mainly independent from HHT disease and described in literature data as potentially contributors of more unfavorable outcome, with 178/296 (60.1%) of them declaring at least one chronic concomitant affection, and 41/296 (13.9%) of them declaring ≥ 3 co-morbidities. Diabetes and hypertension arose as the most frequently concomitant pathologies, occurring in 82/296 (27.7%) and 71/296 (24.0%) cases, respectively.

**Table 1:** HHT-related and –unrelated clinical characteristics in the 296 patients with hereditary hemorrhagic telangiectasia (HHT), who responded to the SarsCov-2 infection questionnaire

*General (n = 296)*	
Age years-old, mean (SD); Range	52.94 (17.59); 3–86
Age class, n (%)	
< 40 years	55 (18.6)
40–64 years	158 (53.4)
≥ 65 years	83 (28.0)
Gender (Female), n (%)	157 (53.0)
*Clinical, HHT-related (n = 296)*^a^	
Epistaxis, n (%)	283/296 (95.6)
PAVMs, n (%)	100/279 (35.8)
BAVMs, n (%)	27/235 (11.5)
HAVMs, n (%)	104/275 (37.8)
GI bleeding, n (%)	52/296 (17.6)
Mutated Gene (n = 296)	
Identified Mutation, n (%)	236 (79.7)
* ENG*	86 (36.5)
* ALK1/ACVRL1*	149 (63.1)
* SMAD4*	1 (0.4)
Mutation unidentified/Ongoing analysis, n (%)	60 (20.3)
*Co-morbidity condition, (n = 296)*	
Absence of Co-morbidity, n (%)	118 (39.9)
Presence of Co-morbidity, n (%)	178 (60.1)
Diabetes, n (%)	82 (27.7)
Hypertension, n (%)	71 (24.0)
Asthma, n (%)	25 (8.4)
History of Stroke, n (%)	8 (2.7)
Smoking, n (%)	43 (14.5)
Obesity, n (%)	33 (11.1)
Renal Insufficiency, n (%)	6 (2.0)
COPD, n (%)	11 (3.7)
Pulmonary Hypertension, n (%)	5 (1.7)
Oncologic Disease, n (%)	10 (3.4)
Other, n (%)	47 (15.9)
< 3 Co-morbidity conditions, n (%)	137 (46.3)
≥ 3 Co-morbidity conditions, n (%)	41 (13.9)

*BAVMs *brain arterio-venous malformations, *HAVMs *hepatic arterio-venous malformations, *PAVMs *pulmonary arterio-venous malformations

^a^The presence of HHT-related visceral arterio-venous malformations was computed by considering only those patients with screening performed in each given organ

### COVID-19 cases

In the first wave, 9/296 (3.04%) HHT patients reported to be affected by an ascertained SarS-Cov-2 infection. The 9 COVID-19 patients had a mean age of 46.22 ± 9.55 years, ranging from 35 to 65 years, with a male-to-female ratio of 2:1 (6 M:3F). Diagnosis was confirmed in all of the 9 cases, by PCR-based molecular assay on Nasopharyngeal/Oropharyngeal Swab, and/or serological test. In details, 4/9 (4.4%) patients had both swab-executed COVID-19 molecular confirmation and serological confirmation, while the remaining 5 patients had only serological confirmation, together with COVID-19-typical clinical manifestations (Table 2). Due to laboratory network overload taking place in Italy during the first wave, two patients were never molecularly tested whilst three cases were molecularly tested only > 2 months after symptom onset, when complete negativization had likely been achieved.

**Table 2:** Baseline characteristics of the 9 patients with hereditary hemorrhagic telangiectasia (HHT) with ascertained SarsCov-2 infection

ID	Gender	Age	Residency region (province)	HHT type	PCR-based molecular diagnosis on swab sample	Nasopharyngeal/oropharyngeal swab	Serol. test
4	F	41	Lombardy (Bergamo)	HHT2	Apparently Negative, but Molecular test was performed three months after symptom onset	Nasal	Pos
20	M	46	Lombardy (Bergamo)	HHT2	Positive	Nasal	Pos
22	M	44	Lombardy (Bergamo)	HHT2	Positive	Nasal	Pos
24	M	57	Liguria (Savona)	HHT2	Positive	Nasal/Oral	Pos
34	F	38	Lombardy (Bergamo)	HHT1	Molecular test never performed^a^	/	Pos
39	F	35	Lombardy (Cremona)	HHT1	Apparently Negative, but molecular test was performed two months after symptom onset	Nasal/Oral	Pos
49	M	41	Lombardy (Bergamo)	HHT2	Apparently Negative, but molecular test was performed two months after symptom onset	Nasal	Pos
66	M	49	Lombardy (Bergamo)	HHT2	Molecular test never performed^a^	/	Pos
119	M	65	Lombardy (Brescia)	HHT1	Positive	Nasal/Oral	Pos

*HHT1* hereditary hemorrhagic telangiectasia type 1, *HHT2* hereditary hemorrhagic telangiectasia type 2, *PCR* polymerase chain reaction

^a^Symptom onset occurring before lockdown/pandemic declaration, when routinely molecular assay on swab sample was not established

Clinical spectrum was variable, ranging from no or minimal symptomatology to intensive-care-requiring very severe manifestations (Table 3). Symptoms associated to the SarS-Cov-2 infection were reported by 8/9 COVID-19 subjects (88.9%), while one patient was completely asymptomatic and caught upon tracing procedure (Table 4). Among the eight symptomatic patients, one subject displayed a very mild expression, consisting in smell/taste loss as the only clinical manifestation. Gustative/olfactory chemosensory impairment was also reported in two more patients, along with other symptoms. Fever was the commonest reported feature, as it was present in 7/9 (77.8%) COVID-19 patients, followed by sense of tiredness/fatigue (5/9 patients), coughing and respiratory dyspnea (4/9 patients each). Headache, muscular pain, diarrhoea, sore throat were also included within the reported symptomatology, whereas none of the respondent suffered from rhinorrhea or conjunctivitis. Multiple symptoms were reported in 7/9 (77.8%) patients, with four of them suffering from > 3 symptoms. Radiological investigations were carried on in 4/9 patients, revealing signs of bilateral interstitial pneumonia in all of them.

**Table 3:** Clinical symptomatology reported by the 9 HHT patients with ascertained SarsCov-2 infection

*COVID-19, ascertained (n = 9)*	
Age years-old, mean (SD)	46.22 ± 9.55
Range	35–65
Gender (Female), n (%)	3 (33.3)
*Clinical symptomatology, COVID-19*	
Fever, n (%)	7/9 (77.8)
Coughing, n (%)	4/9 (44.4)
Headache, n (%)	2/9 (22.2)
Dyspnea, n (%)	4/9 (44.4)
Muscular Pain, n (%)	3/9 (33.3)
Sense of Tiredness/Fatigue, n (%)	5/9 (55.5)
Diarrhoea, n (%)	2/9 (22.2)
Sore Throat, n (%)	2/9 (22.2)
Rhinorrhea, n (%)	0/9
Conjunctivitis, n (%)	0/9
Gustative/olfactory sensory impairment, n (%)	3/9 (33.3)
None (Asymptomatic), n (%)	1/9 (11.1)
*Clinical management and outcomes, COVID-19*	
Rx-confirmed Pneumonia^a^, n (%)	4/9 (44.4)
Domiciliar management, n (%)	7/9 (77.8)
Oxygen support therapy at domiciliar regimen, n (%)	0/9
Hospitalization required, n (%)	2/9 (22.2)
Non-invasive ventilation, n (%)	1/9 (11.1)
Invasive ventilation (intubation), n (%)	1/9 (11.1)
Death, n (%)	0/9

^a^Chest radiological investigation was performed in 4 patients only

**Table 4:** Clinical characteristics of the 9 patients with hereditary hemorrhagic telangiectasia (HHT) with ascertained SarsCov-2 infection

ID	Gender	Age	Epistaxis before SarsCov-2 infection	Epistaxis profile during infection	Visceral involvement (AVMs)	Hb deficit level	Covid-19 symptoms/manifestations	Non-HHT co-morbidities
4	F	41	Yes	Worsening	No	Mild	GOS	Osteoporosis, Autoimmune Thyroiditis
20	M	46	Yes	Unchanged	No	Mild	Asymptomatic	Hypert., Hemodialysis, Chronic Kidney Disease
22	M	44	Yes	Unchanged	No	Intermediate	Pn, F, C,T, R, D, ST, GOS^a^	
24	M	57	Yes	Unchanged	No		Pn, F, C, R^b^	
34	F	38	Yes	Worsening	No		F, R, M,T, ST	
39	F	35	Yes	Worsening	P, H	Mild	F, H, M,T, D	Asthma
49	M	41	Yes	Unchanged	No	Mild	F, C,H, M,T,GOS	
66	M	49	Yes	Unchanged	No		Pn, F, R	Hypert
119	M	65	Yes	Unchanged	P, H, GI	Mild	Pn, F, C, T	

Visceral Involvement: Organ displaying HHT-related Arterio-Venous Malformations (AVMs). *P *pulmonary arterio-venous malformations, *H *hepatic arterio-venous malformations, *GI *gastrointestinal arterio-venous malformations, *Pn *pneumonia, *F *fever, *C *coughing, *H *headache, *R *respiratory dyspnea, *M *muscular pain, *T *sense of tiredness/fatigue, *D *diarrhoea, *ST *sore throat, *GOS *gustative/olfactory sensory impairment. Hb level: Mild (Hb > 12 gr/dl), Intermediate (10–12 gr/dl), Severe 7–10 gr/dl), Very Severe (< 7 gr/dl)

^a^Needed Hospitalization and Intensive care (Invasive Ventilation)

^b^Needed Hospitalization and Intensive care (Non-Invasive Ventilation)

The majority of patients (7/9, 77.8%), including the asymptomatic patient, the anosmia/ageusia-only patient, and five of the patients affected by fever and other manifestations, had a benign clinical course, resolving by home management without either major complications or oxygen support therapy. However, 2/9 individuals (22.2%) experienced a more aggressive clinical profile requiring hospitalization and intensive care management. Severe lung disease arose in a 57-year-old patient, with consequent hypoxemia, successfully treated with C-PAP non-invasive ventilation. A 44-year-old patient developed an even more serious and life-threatening bilateral interstitial pneumonia, which regressed only after invasive mechanical intubation, ultimately leading to hospital discharge. Both severe cases were male patients. At the end of the study, the disease entailed no sequelae in all patients, except for the intubation-requiring 44-year-old patient, who reported persistent post-discharge dizziness episodes, despite recovering from lung disease.

An additional seven patients (7/296, 2.69%) reported a suspected SarS-Cov-2 infection at questionnaire survey (mean age 45.86 ± 21.45 years, range 3–70 years, female gender 5/7, 71.4%). PCR-based molecular assay on Nasopharyngeal/Oropharyngeal Swab was performed in 5/7 cases (71.4%) and yielded a negative results in all of them, with three of them having molecular testing performed > 2 months after symptom resolution. None of the patients needed hospitalization or required oxygen supplementation, with complete domiciliary resolution in all patients.

Overall, a total of 16/296 (5.40%) HHT patients reported an ascertained or suspected SarS-Cov-2 infection. However, in order to minimize inclusion bias, only cases with ascertained SarS-Cov-2 infection diagnosis were included in further statistical and epidemiological analyses.

### COVID-19 and HHT-related bleeding

All of the 9 COVID-19 patients had suffered from long-lasting HHT-related epistaxis before infection (Table 4). Worsening in epistaxis severity was reported in 3/9 (33.3%) patients. No correlation was found between nosebleeds increase and severe COVID-19 features, as such complaint was not reported by the two hospitalized patients, while it was only recorded by three individuals characterized by domiciliary resolution. Three patients were also able to report their sensation of subjective status concerning their nose, with increased dryness reported by two patients and increased wetness by one. On the other hand, none of the respondents reported any changes in bleeding from oral telangiectases.

### COVID-19 susceptibility and HHT-related manifestations

The 9 ascertained COVID-19 patients had no statistically significant difference in age distribution when compared to COVID-19-free patients. Upon stratifying the cohort according to age class (< 40 years, 40–64 years, ≥ 65 years), the proportion of infected individuals showed no statistically significant difference (2/55, 3.6%; 6/158, 3.8%; 1/83, 1.2%, respectively, *p* = 0.591). In the 9 ascertained COVID-19 patients, the HHT-causing mutation was located in the *ENG* gene (HHT1) and in the *ALK1/ACVRL1* gene (HHT2) in 3 and 6 patients, respectively, thus resulting in an overlapping distribution with the COVID-19-free patients. HHT-related visceral involvement was reported in 2/9 (22.2%) ascertained COVID-19 patients (Table 4). When the nine COVID-19 patients were compared with COVID-19-free patients, no statistically significant difference was observed in terms of presence of nosebleeds, pulmonary AVMs, brain AVMs, hepatic and GI involvement, or non-HHT-related illness (Table 5).

**Table 5:** Comparison between HHT patients with ascertained SarsCov-2 infection vs. COVID-19-free patients

	Ascertained COVID (n = 9)	Non COVID (n = 287)	*p* value
*General (n = 296)*			
Age years-old, mean (SD)	46.22 (9.55)	53.15 (17.78)	0.25
Range	35–65	3–86	
Gender (female), n (%)	3 (33.3)	154	0.31
*Clinical, HHT-related (n = 296)*^a^			
Epistaxis, n (%)	9/9 (100)	274/287 (95.5)	1
PAVMs, n (%)	2/9 (22.2)	98/270 (36.3)	0.5
BAVMs, n (%)	0	27/226 (11.9)	0.4
HAVMs, n (%)	2/9 (22.2)	102/266 (38.3)	1
GI bleeding, n (%)	1/9 (11.1)	51/287 (17.8)	1
*Mutated gene (n = 296)*			
Identified Mutation, n (%)	9 (100)	227 (79.1)	0.13
* ENG*	3 (33.3)	83 (29)	
* ALK1/ACVRL1*	6 (66.7)	143 (49.8)	
* SMAD4*	0	1 (0.3)	
Mutation unidentified/Ongoing analysis, n (%)	0	60 (20.9)	

^a^The presence of HHT-related visceral arterio-venous malformations was computed by considering only those patients with screening performed in each given organ

*BAVMs *brain arterio-venous malformations, *HAVMs *hepatic arterio-venous malformations, *PAVMs *pulmonary arterio-venous malformations

### COVID-19 outcome and HHT-related manifestations

We tried to gain insight into the potential effect of HHT-related features on the clinical trajectory during SarS-Cov-2 infection (Tables 4, 6). Both of the patients with HHT-related visceral involvement had previously successfully treated pulmonary AVMs, as well as silent hepatic AVMs, one of them also suffering from GI involvement. Nonetheless, they experienced a benign profile of their clinical course, with uneventful domiciliary management. On the opposite, no visceral AVMs was present in the two intensive-care-requiring patients.

**Table 6:** Clinical HHT-related features of the 9 patients with hereditary hemorrhagic telangiectasia (HHT) with ascertained SarsCov-2 infection

*Clinical, HHT-related (n = 9*^a^*)*	
Epistaxis, n (%)	9 (100)
Visceral AVMs, n (%)	2 (22.2)
Epistaxis Worsening, n (%)	3 (33.3)
Epistaxis Unchanged, n (%)	6 (66.7)
Epistaxis Improvement, n (%)	0
Increased bleeding from oral telangiectases, n (%)	0
*Chronic Anemia*	
Hb deficit level (n = 6^a^)	
Mild, n	5
Intermediate, n	1
Severe, n	0
Very Severe, n	0
Iron replacement therapy (n = 8^a^)	
Oral Supplementation, n	3
IV Injection, n	2
Blood Transfusion, n	0

AVMs = Arterio-Venous Malformations; Hb level deficit: Mild (Hb > 12 gr/dl), Intermediate (10–12 gr/dl), Severe 7–10 gr/dl), Very Severe (< 7 gr/dl); ^a^Number of respondents for each variable

Among the 6 patients able to recall their Hb levels, five of them reported mild Hb deficit levels (> 12 gr/dl) and one of them reported intermediate Hb levels (10–12 gr/dl), while none of them reported severe or very severe anemia (Table 6). Five patients reported iron supplementation therapy before infection occurrence, with no relevant changes needed throughout COVID-19 course, including the 57-year-old patient requiring non-invasive ventilation. Noteworthy, the only patient reporting intermediate Hb levels was the 44-year-old patient needing intubation, who was not on replacement therapy, despite his anemia condition.

Likewise, no clear role was evident between COVID-19 clinical course and non-HHT-related co-morbid conditions, as no concurrent pathologies were reported by the two intensive-care-requiring patients (Table 4).

### COVID-19 prevalence

In order to estimate the risk to develop COVID-19 among the HHT population, we tried to compare our data with the general Italian population during the first wave. In our overview, the raw prevalence of ascertained SarS-Cov-2 infection among HHT patients was 9/296 (3.04%).

Since the distribution of COVID-19 cases showed a marked geographical dishomogeneity in Italy, throughout the first wave [33, 34], we decided to normalize our results, with respect to the Region and Province of patient’s actual residency during the study period. As shown in Table 2, all of the 9 COVID-19 patients resided in Northern Italy, which was more deeply involved by the first pandemic wave, and 8 out of the 9 patients were resident in Lombardy, the region where the first European autochthonous cause of COVID-19 was identified [33]. Furthermore, a deeper insight disclosed that the Province of residency of the 8 Lombard COVID-19 patients coincided with the three Provinces characterized by the highest SarS-Cov-2 infection concentration, as representing the very first and dramatic epidemic foci (6 cases from Bergamo, 1 case from Brescia, 1 case from Cremona, respectively, Tables 1, 7) [35]. Therefore, we computed the observed raw prevalence values by focusing analysis on Lombardy Region only. Table 8 displays prevalence data, as obtained when analysis was corrected to account for geographical residency, respondents’ group population, and molecular vs. serological confirmation. We found a prevalence as high as 8/63 (1:7.87; 12.7%, 95% CI 5.6–23.5%) for whole Lombardy Region, a seemingly much higher rate than the cumulative value officially reported for whole Lombard population on the end study date (2020, June 30th), equal to 1:107, the difference being statistically significant (*p* = 0.002). In order to further refine our epidemiological analysis, we speculated on potential bias in respondents’ population, thus expanding the denominator by also considering the 68 Lombard non-respondents as the susceptible population. Since the number of eligible Lombard patients contacted for the present survey was 131, the prevalence would drop to 8/131 (1:16.37; 6.10%, 95% CI 2.7–11.7%), with the 8 HHT Lombard cases still including all serologically-confirmed cases, regardless of the presence of molecular confirmation. Moreover, when analysis was restricted only to HHT cases with molecularly-confirmed SarS-Cov-2 infection, thus fulfilling homogeneity between Lombard study population and reference population, the prevalence rate value pointed to a value of 3/131 (1:43.67; 2.29%, 95%CI 0.5–6.5%), not significantly different from the general Lombard population (*p* = 0.10), thus suggesting that the presence of HHT itself does not necessarily entail an increased relative risk to contract SarS-CoV-2 infection compared to general population (2.44 RR, 95% CI 0.80–7.49).

**Table 7:** Raw prevalence rates of COVID-19 cases among Lombardy general population, stratified by Province

Province	Observed raw prevalence rate in general population^a^
**Lombardy region**	**1:107**
Cremona	1:54
Lodi	1:64
Bergamo	1:77
Brescia	1:80
Pavia	1:97
Sondrio	1:114
Mantova	1:116
Lecco	1:118
Milano	1:134
Como	1:146
Monza Brianza	1:151
Varese	1:226

Whole Lombardy Region in bold, single provinces in plain text

Lombardy Region: Total general population, 2020: **10,027,602** (ISTAT, 2019, Dec 31st). Number of COVID-19 cases reported at the end of study period (2020, June 30th): **93,901**

^a^Prevalence rate at the end of study period (2020, June 30th)

**Table 8:** Comparison between raw prevalence rates of COVID-19 cases among Lombardy general population and Lombardy population with HHT

Lombardy region					
*General population*					
Susceptible Population^a^				COVID-19 positive in general population Only cases with swab molecular confirmation^b^	Observed Raw prevalence rate in general population Only cases with swab molecular confirmation^b^
**10,027,602**				93,901	1:107
*HHT*					
	Observed COVID-19 positive cases in HHT	Observed Raw prevalence rate in HHT	Observed Raw prevalence rate in HHT	Observed COVID-19 positive cases in HHT	Observed Raw prevalence rate in HHT
	Serol. confirmation, irrespective of swab molecular confirmation^b^	Serol. confirmation, irrespective of swab molecular confirmation^b^ **(Susceptible population:** Respondents only = **63)**	Serol. confirmation, irrespective of swab molecular confirmation^b^ **(Susceptible population:** Non-Respondents included = **131)**	Only cases with swab molecular confirmation^b^	Only cases with swab molecular confirmation^b^ **(Susceptible population:** Non-Respondents included = **131)**
	8	8/63 (12.7%) 1:7.87	8/131 (6.1%) 1:16.37	3	3/131 (2.29%) 1:43.67

Susceptible population is indicated in bold

(^a^ISTAT, 2019, Dec 31st) (Lombardy population, 2020)

^b^Cases reported at the end of study period: (2020, June 30th)

### Effect of personal protective equipment/individual protection devices

A total of 294 patients responded to the questions concerning wearing of PPE/DPI. The vast majority of respondents (289/294, 98.3%) stated they wear facemasks during study period (surgical or homemade masks, professional FFP2, professional FFP3). Two-hundred and ten patients reported employment of one face PPE/DPI type only, while the remaining 79 employed ≥ 2 PPE/DPI types. Complications/annoyances (bleeding nasal or oral telangiectases) were noticed by 42/289 (14.5%) facemask-wearing individuals, with no significant difference in terms of number or type of utilized PPE/DPI. Adopting hand hygiene measures was reported by 288/294 (98%) individuals, with four of them suffering from cutaneous telangiectasia bleeding as a complication.

## Discussion

COVID-19 disease, emerging in Wuhan, China, in December 2019, is a pandemic dramatic condition that has changed the world in many ways. From the emerging literature, older age, and the presence of a number of underlying health conditions, such as cardiovascular diseases, diabetes, or other chronic pathologies, seem to have an increased risk of an adverse COVID-19 outcome, including hospitalisation and mortality [36]. Hence, characterizing the contributions of specific disease phenotypes to SarS-CoV-2 susceptibility and risk of adverse poor outcomes is essential for effective policy-making and healthcare planning, as well as to optimize efficacy of ongoing vaccination campaign [37]. However, determining the influence of RDs on COVID-19 increased risk and/or outcomes severity is often limited by the rarity of the condition and the consequent need for a wide baseline study population [6]. In this respect, the extensive outbreak growth in Italy, the first Western country to face a huge number of COVID-19 patients [38], resulting in 238,082 cases and 33,209 deaths at the end date of the present study, expanded the possibility of large-scale clinical case reporting, thus providing the possibility of population-level snapshots of the impact of RDs on COVID-19 course.

In the present study, we report for the first time the results of a nation-wide epidemiologic survey, aimed to assess the prevalence of SarS-CoV-2 infection and to characterize the clinical course of COVID-19 disease in a population with HHT, an autosomal-dominantly-inherited RD. Several points of concern can be raised when considering the infection susceptibility of HHT patients compared to general population. HHT patients might in fact be characterized by heightened infection risk, mainly due to repeated manipulation of nasal airways needed for nosebleeds self-management and frequent contacts with healthcare personnel within hospital setting for various reasons, including uncontrollable epistaxis, sudden complications secondary to visceral AVMs, or regular treatments of chronic iron-deficiency anemia [20]. We found a prevalence of 9/296 (3.04%) of ascertained COVID-19 disease among Italian population during the first wave. Although COVID-19 diagnosis was suspected in seven additional patients, we did not include them in epidemiological analysis, in order to avoid inclusion of potential false positive cases. We emphasize herewith that we decided to adopt a very strict case definition, in that only patients with laboratory test confirmation (either molecular PCR-based or serological) were considered as “ascertained COVID-19 cases”, which permitted to strengthen the relevance of our results, also in light of future epidemiological comparisons with studies in other populations.

The main finding of our study is that infection spread in HHT population seemed to parallel the geographical distribution of epidemic profile occurring across Italy throughout the first wave. All of the nine ascertained COVID-19 patients resided in Northern Italy, which were faced by the highest prevalence rates during the first wave, mainly concentrating around an area in the Lombardy Region, corresponding to the epicenter of pandemic outbreak [35, 39, 40]. Rather, it is conceivable that patients from Central and Southern regions benefited from reduced contact frequency, similar to the general population’s behavior, as a result of implementation of restriction measures in earlier phases of epidemic spreading [33]. This observation suggests that future epidemiological estimates regarding COVID-19 prevalence should always be normalized according to geographical fluctuations due to epidemic foci distribution.

Indeed, there is unprecedented urgency to understand whether HHT represents an underlying pathology characterized by increased susceptibility to COVID-19 infection compared to general population, an issue that we tried to address by restricting analysis to Lombardy residents only. The second major finding of the present study represents the first estimated population-based prevalence rate of COVID-19 among HHT subjects. Initial gross estimates seemed to suggest a markedly higher frequency in HHT vs general population (1:7 vs. 1:107), which nonetheless progressively approached values closer to the expected value when epidemiological analysis was refined (1:43 vs. 1:107), with no clear evidence of heightened relative risk of infection profile (2.44 RR, 95%CI 0.80–7.49). The progressive convergence of the HHT prevalence rate towards the general population rate that we observed can have several explanations, including an underestimation of molecularly-confirmed cases in general population, as described by multiple reports [32, 39], and an overestimation of cases in the HHT population, due to an enrichment bias in the respondents’ sample. Throughout the first wave in Italy, dramatic overstretching of laboratory resources and tracing systems led to a considerable case underascertainment, especially in those Regions mostly involved by the epidemics [39, 41]. Consistent with this scenario, 5/9 patients reported absence of timely molecularly testing on swab sample in our study. Secondly, although we cannot completely rule out the possibility of COVID-19 cases among non-respondents, it is plausible that HHT patients with history of COVID-19 infection were presumably more alerted to respond the questionnaire and be recruited in the study. Whilst the true prevalence of molecularly-confirmed cases in general population in Italy’s first wave is unknown, preliminary seroprevalence data suggest a value of 7.5% (95% CI 6.8–8.3%) for Lombardy region [42]. Noteworthy, this interval overlaps to the prevalence value of COVID-19-infected individuals among HHT observed in this study for the Lombardy region, arising when all serologically-positive COVID-19 cases are considered irrespective of molecular confirmation, and when the non-respondents are included among the susceptible population, namely 8/131 (1:16; 6.10%, 95% CI 2.7–11.7%), which strongly support our findings. In a very recent survey carried on in Spain, the seemingly higher COVID-19 prevalence in HHT patients compared to general population was attributed to an augmented propensity to rate of testing in HHT patients [43]. Accordingly, we clearly provided evidence herein that the apparent increased COVID-19 frequency in HHT seems to lack consistency, once ascertainment bias associated to epidemiological distribution are properly corrected for. An alternative explanation might also be evoked for the interpretation of our results, considering the hypothesis that HHT could have potentially conferred a higher infection risk than that of general population, which was, nonetheless, somewhat counterbalanced by a better propensity of HHT patients to minimize contact frequency in various ways. Patients’ awareness of living with a RD, potentially implying a worst outcome, might have strengthened compliance to lockdown and social distancing, even though we were not able to address this point, which requires comparison of contact frequency of HHT vs. non-HHT individuals in non-lockdown conditions.

Data reported herewith provide, for the first time, observations regarding the range of the possible clinical evolution and outcomes in HHT individuals with SarS-COV-2 infection. Our study provides evidence that the whole COVID-19 spectrum can be displayed by HHT patients, ranging from completely asymptomatic, anosmia/ageusia only, fever and influenza-like symptoms, to interstitial pneumonia which, in a few cases, became prone to clinical deterioration. Despite respiratory failure prompted need for mechanical ventilation and intensive care admission in two cases, it is paramount to underline that no fatal outcome occurred. As COVID-19 spreading rapidly exceeds hospital capacities, the decision between hospitalization or domiciliary management is crucial to relieve hospitals from overload [44]. Additionally, limited resource allocation poses a challenging issue in terms of clinical decision-making, in contexts where intensive care admission entails priority scores based on age, survival prognosis and pre-existing illnesses [45]. HHT is a rare disease potentially involving chronic conditions with a reduced life-expectancy, especially in absence of appropriate pre-emptive management [46–48]. Our data clearly indicate that most HHT patients have quite a benign course, with uneventful domiciliary management without need of hospitalization. Moreover, the favorable outcome of the two hospitalized patients permits to emphasize that the presence of HHT as a pre-existing pathology should not imply a lower priority score than the general population, whenever access to intensive care, including invasive ventilation, is needed.

Our analysis did not reveal any HHT-related or un-related features predisposing HHT patients to increased infection risk. Similarly, the patients with the worst outcome did not differ significantly from those experiencing a more favorable disease course. These observations seem to suggest that susceptibility infection profile and poor outcome risk in these patients may parallel the scenario in the general population, as already suggested by the HHT working group of the European Reference Network for Rare Multisystemic Vascular Diseases (VascERN) [49]. However, some HHT-related chronic complications, such as PAVM-related respiratory insufficiency, brain abscess sequelae, chronic iron-deficiency anemia, HAVM-related high output cardiac failure might actually represent a subgroup of heightened risk of COVID-19-associated adverse outcome events. None of the COVID-19 patients identified in the present study suffered from these serious complications, which prevented us from addressing this issue. Even though a weaker severity of infection symptomatology has been hypothesized in HHT patients, in terms of hospitalization rate and need for ventilation [43, 50], potentially attributing to reduced endothelial damage and/or impaired inflammatory cytokine profile, our result do not seem to support this scenario, as 2/296 interviewed patients were hospitalized for COVID-19 pneumonia across Italy, also requiring ventilatory support or intubation. In HHT, Riera-Mestre et al. [50] have reported a low hospitalization prevalence for COVID-19 pneumonia (1/1177), and Marcos et al. [43] have observed a mild degree of manifestations in all patients affected by COVID-19 (only 3/138 individuals required hospitalization, none of them needing ventilation or intensive care). Discrepancy can be accounted for by the different approach of our study, which aimed to fully characterize the clinical spectrum of HHT patients with COVID-19 and to weigh the results according to the geographical distribution of epidemic foci, in a nation-wide manner, whereas focusing only on COVID-19-pneumonia-related hospital admission [50], or investigating a single cohort attending a reference HHT ENT consult [43], without normalizing for epidemiological profile, might result in an incomplete description of COVID-19 clinical array in a rare disease as HHT. Interestingly, the patient experiencing the worst outcome was the only one who reported intermediate Hb level deficit throughout the infection, which could have increased his vulnerability to the augmented demand posed by the infection, also in light of his lacking proper correction of anemia condition. As a single observation needing confirmation in future studies, such hypothesis should be regarded as a speculation, although we feel to advocate appropriate iron supplemental therapy maintenance in pandemic condition, as already evidenced [49]. Finally, further investigations are needed to better characterize the contribution of advanced age, a well-known predictor of severe clinical evolution, to the clinical picture of COVID-19 in HHT, as only one affected individual belonging to the geriatric age class (≥ 65 year-old) was captured in the present study. However, no statistically significant difference was found in the proportion of affected individuals among the < 40 years, 40–64 years, and ≥ 65 years age subgroups, and the percentage of ≥ 65 year-old subjects in our cohort is not lower than in Italian general population (28% vs. 23.5%, respectively, [51]), suggesting that our results were not substantially affected by under-representation of elderly individuals in our cohort.

Our study has some limitations. As in many investigations concerning RDs, the retrospective study design may not permit to address all clinically relevant data, due to the reduced sample size and the limited observational period. The study was carried out as a questionnaire-based inquiry, which may result in limited accuracy of clinical information, based on patient’s recall rather than on clinical charts. Furthermore, no severe HHT-related manifestations/complications characterized the clinical baseline framework of the COVID-19 patients before infection, thus suggesting that future studies are needed to assess the potential contribution of serious HHT-related features to COVID-19 outcome profile.

## Conclusions

Sars-CoV-2 infection profile parallels geographical distribution of epidemic foci. COVID-19 in HHT patients can lead to highly variable clinical manifestations, with a clinical spectrum which appears to overlap with that of general population. The HHT disease does not seem to involve a different approach in terms of hospital admission and access to intensive care with respect to general population. Future studies will assess the impact of HHT-related chronic complications on outcome.

## Data Availability

The data that support the findings of this study are not publicly available, due to individual privacy reasons, as they contain information that could compromise questionnaire respondents’ privacy.

## Abbreviations

COVID-19: coronavirus disease 2019
SarS-CoV-2: severe acute respiratory syndrome coronavirus 2
RD: rare diseases
HHT: hereditary hemorrhagic telangiectasia
AVM: arterio-venous malformation
PPE/DPI: personal protective equipment/individual protection devices
PCR: polymerase chain reaction
BAVM: brain arterio-venous malformation
HAVM: hepatic arterio-venous malformation
PAVM: pulmonary arterio-venous malformation
Pn: pneumonia
F: fever
C: coughing
H: headache
R: respiratory dyspnea
M: muscular pain
T: sense of tiredness/fatigue
D: diarrhoea
ST: sore throat
GOS: gustative/olfactory sensory impairment
